# NIOSH Dampness and Mold Assessment Tool (DMAT): Documentation and Data Analysis of Dampness and Mold-Related Damage in Buildings and Its Application

**DOI:** 10.3390/buildings12081075

**Published:** 2022-07

**Authors:** Ju-Hyeong Park, Jean M. Cox-Ganser

**Affiliations:** Respiratory Health Division, National Institute for Occupational Safety and Health Centers for Disease Control and Prevention, Morgantown, WV 26505, USA;

**Keywords:** dampness and mold, DMAT, indoor environments, observational assessment, visual and olfactory assessment, data analysis and application, water damage

## Abstract

Indoor dampness and mold are prevalent, and the exposure has been associated with various illnesses such as the exacerbation of existing asthma, asthma development, current asthma, ever-diagnosed asthma, bronchitis, respiratory infection, allergic rhinitis, dyspnea, wheezing, cough, upper respiratory symptoms, and eczema. However, assessing exposures or environments in damp and moldy buildings/rooms, especially by collecting and analyzing environmental samples for microbial agents, is complicated. Nonetheless, observational assessment (visual and olfactory inspection) has been demonstrated as an effective method for evaluating indoor dampness and mold. The National Institute for Occupational Safety and Health developed an observational assessment method called the Dampness and Mold Assessment Tool (DMAT). The DMAT uses a semi-quantitative approach to score the level of dampness and mold-related damage (mold odor, water damage/stains, visible mold, and wetness/dampness) by intensity or size for each of the room components (ceiling, walls, windows, floor, furnishings, ventilation system, pipes, and supplies and materials). Total or average room scores and factor-or component-specific scores can be calculated for data analysis. Because the DMAT uses a semi-quantitative scoring method, it better differentiates the level of damage compared to the binary (presence or absence of damage) approach. Thus, our DMAT provides useful information on identifying dampness and mold, tracking and comparing past and present damage by the scores, and prioritizing remediation to avoid potential adverse health effects in occupants. This protocol-type article describes the DMAT and demonstrates how to apply it to effectively manage indoor dampness and mold-related damage.

## 1. Introduction

Water leaks, construction faults, indoor condensation, the malfunction of ventilation systems, or flooding from extreme weather events could lead to damp indoor environments under which microbes can proliferate as available water increases in building materials [1,2]. Although there is no absolute definition of dampness and mold (hereafter, dampness/mold), it commonly includes four water-damage-related aspects such as visible wetness or dampness, water damage or stains, visible mold, and mold odor [3,4]. The prevalence of dampness/mold in indoor environments varies widely (10–50%), depending on continents, countries, and regions within countries [2]. The most recent 2019 American Housing Survey of the U.S. Census indicated that 9.4% of U.S. homes had exterior water leakage and 7.6% had interior water leakage [5]. A study of 831 residential homes from 75 different locations in the U.S. reported that 24% of the surveyed homes had moisture or mold problems [6]. Although there are no national data on the prevalence of dampness/mold in U.S. residential buildings, the population-weighted average prevalence of dampness/mold estimated from several published studies was 47% [7]. There is also a lack of recent national data on the prevalence of dampness/mold for schools and other types of non-residential buildings. The longstanding 1995 U.S. General Accounting Office (currently, General Accountability Office) survey on school buildings indicated that 30% of schools in the U.S. had plumbing problems and 27% had roofing problems that could lead to interior or exterior water leakage [8]. The U.S. EPA (Environmental Protection Agency) BASE (Building Assessment Survey and Evaluation) study of 100 randomly selected public and commercial office buildings across the U.S. conducted during 1994 through 1998 showed that 85% of the buildings experienced past water damage and 45% had current leakage problems [9,10].

On the other hand, a study of the prevalence of dampness/mold in homes in 31 European countries based on 16 individual surveys and three large multinational datasets indicated that the weighted prevalence was 12% for dampness or condensation, 10% for visible mold, 10% for water damage, and 17% for any one of these dampness/mold-related factors [11]. The European Community Respiratory Health Survey (ECRHS) II reported that the prevalence of self-reported water damage was 28%, while the prevalence of self-reported visible mold was 25%, and visible mold was observed in 14% of European homes [12]. A study of schools in three European countries (the Netherlands, Spain, and Finland) reported that 20–41% of the school buildings (differed by country) had moisture problems [13]. Another study of all types of non-industrial occupational buildings from the ECRHS II survey reported 11% of the surveyed workplace buildings had water leakage, signs of floor dampness, visible mold, or moldy odors [14].

In damp indoor environments, occupants can be exposed to elevated levels of various airborne microbial agents. These could include intact bacterial or fungal cells, cell fragments, or their cell wall components such as endotoxin (a cell wall component of Gram-negative bacteria), ergosterol (a cell wall component of fungi), and (1 → 3)-β-D-glucan (a cell wall component of fungi) [1,2,15,16]. Although there are many suggested sampling and analytical methods to measure these airborne microbial agents [17], accurately quantifying them for an epidemiologic study can be complicated, especially for assessing exposure to the potentially harmful agents responsible for targeted health outcomes [2,18–20]. The complexity stems from challenges with the exposure assessment of indoor microbial agents. These include, but are not limited to, the following circumstances: (1) there could be multiple agents causally associated with a specific respiratory symptom or illness from simultaneous exposure to their mixture; (2) it is difficult to accurately quantify airborne concentrations with the limited number of samples and limited resources because of large indoor temporal and spatial variations; (3) the health effects from the exposure to the same agent could be either adverse or beneficial, depending on the timing and dose of exposure; (4) there has been a lack of reliable measurement methods for personal exposure to microbial agents; and (5) there are no established standard or consensus methods for quantifying these agents [2,21–24]. Such complex situations have resulted in rather inconsistent associations between quantitative microbial measurements and respiratory health in many epidemiologic studies of indoor environments [22,23,25]. Unfortunately, these limitations remained mostly unresolved, and thus causal agents for specific health outcomes were not identified until recently [22].

In contrast, the observational assessment of dampness/mold in buildings has been consistently demonstrated to be an effective metric for both the environmental and exposure assessment of dampness/mold-related agents in a large body of scientific literature [1,2,4,23]. The Institute of Medicine (IOM, currently the National Academy of Medicine) and the World Health Organization (WHO) comprehensively reviewed the eligible articles and published their findings in 2004 and 2009, respectively [1,2]. Subsequent articles, published in *Environmental Health Perspectives* in 2011 and 2015, updated the IOM and WHO’s conclusions through the meta-analysis [4] or literature review of newly published studies [26]. These reports and articles concluded that there is sufficient evidence of a causal association of exposure to indoor dampness or dampness-related agents with an exacerbation of existing asthma in children [26]; sufficient evidence of an association with an exacerbation of existing asthma, asthma development, current asthma, ever-diagnosed asthma, bronchitis, respiratory infection, allergic rhinitis, dyspnea, wheezing, cough, upper respiratory symptoms, and eczema; and limited or suggestive evidence of an association with the common cold and allergy/atopy [1,2,4,26]. In addition, Mudarri and Fisk’s report indicated that 20% (95% confidence interval: 12–29%) of current U.S. asthma cases were attributable to indoor dampness/mold exposure and that the related annual cost was USD 3.5 billion [27]. In Europe, about 15% of new childhood asthma and 5–15% of new adult asthma was estimated to be attributable to home dampness/mold [11,12]. All these findings clearly indicate that persistent dampness/mold or microbial growth is a public health hazard that should be prevented; nevertheless, if it occurs, it should be promptly remediated to minimize occupants’ exposure to microbial agents before the damage becomes severe [1,2].

To help facility managers or industrial hygiene (IH) practitioners effectively evaluate water-damage-related indoor environments or assess occupants’ exposure to dampness/mold, the National Institute for Occupational Safety and Health (NIOSH) developed the NIOSH Dampness and Mold Assessment Tool (DMAT). The DMAT is a method that allows users to assess damp indoor environments using observational (such as visual or olfactory) assessment without collecting any environmental samples. This article explains the scientific foundation of the NIOSH DMAT [28], which was published in December 2018, and how to use it, document and analyze the collected data, and apply the results of the data analyses.

## 2. Experimental Design (Scientific Foundation for the Development of the DMAT)

**Figure 1.**
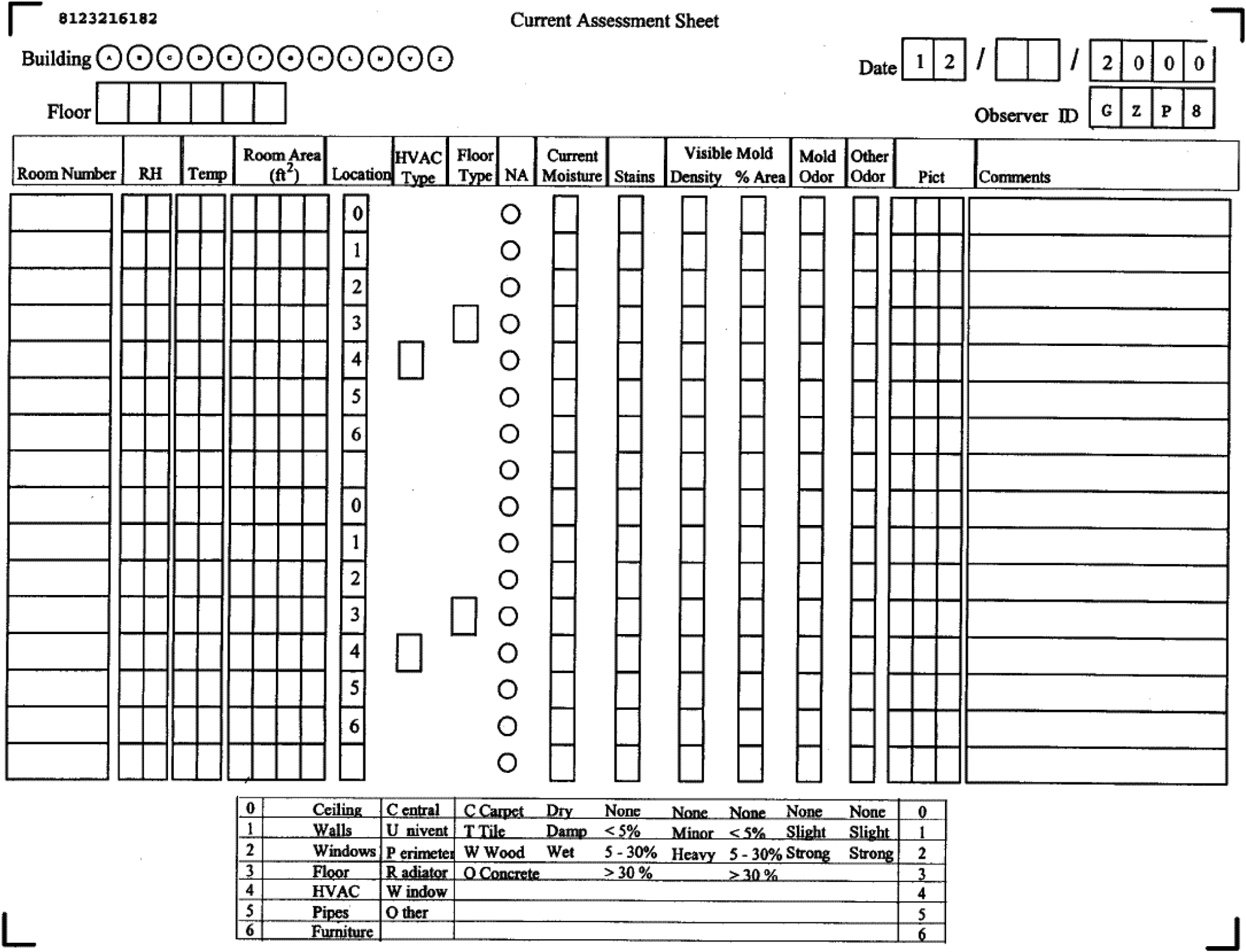
Dampness and mold assessment sheet (a prototype of the DMAT) for the community college study.

We conducted a community college study in New York [29] and a hospital study in Montana [30,31] in the early 2000s. These two studies motivated the development of the DMAT. The community college study investigated 721 rooms from seven water-damaged buildings (558 rooms) and six comparison buildings with no known water damage (163 rooms) [29]. Three teams of two trained industrial hygienists used a prototype of the DMAT for the study, which was a standardized evaluation form (Figure 1). Before the teams started to evaluate the rooms in the buildings, all three teams independently evaluated the same eight rooms in the water-damaged buildings for water stains, visible mold, mold odor, and moisture. They crosschecked their observations among the teams and standardized their assessment methods to ensure that all teams used the same observational methods for evaluating dampness/mold-related factors. Concordance rates were 88% for water stains, 63% for visible mold, 75% for mold odor, and 100% for moisture [29].

As shown in Figure 1, water stains and visible mold were graded on a scale of 0–3 for each room component (denoted as location in the form in Figure 1), based on the percentage of damaged area within the component. Information on the intensity (none/minor/heavy) of visible mold was also collected. Moisture was graded as dry (0), damp (1), or wet (2), and mold odor was graded as none (0), slight (1), or strong (2). This evaluation was systematically performed for each of the seven locations (components) of the room: the ceiling, walls, windows, floor, HVAC (heating, ventilation, and air conditioning) system, water pipes, and furniture. Among the assessed rooms in the water-damaged buildings, water stains were the most prevalent sign of damage (98%), followed by visible mold (20%), mold odor (7%), and moisture (<1%). The room average water-stain score over room components was significantly (*p*-value < 0.05) higher (0.8) in the damaged buildings than the comparison buildings (0.4), and visible mold and mold odor were significantly more prevalent in the damaged buildings (20% and 5%, respectively) than the comparison building (3% and 1%, respectively).

Independent of the environmental survey, a health survey using a questionnaire collected information on respiratory illnesses from 393 participants. For epidemiologic analyses in the community college study, we calculated the individual exposure index to dampness/mold by estimating the time-weighted average dampness score from all rooms where the survey participants spent their time during the semester [29]. From the analyses, we found that the individual exposure index was significantly associated with building-related respiratory symptoms (wheezing, chest tightness, shortness of breath, nasal and sinus symptoms, and throat irritation that improved away from work) in an exposure–response relationship (range of adjusted odds ratios = 1.7–4.4, depending on the health outcome and the type of exposure index). This study demonstrated the utility of the method not only for environmental evaluation but also for exposure assessment in epidemiologic studies.

The hospital study [30] evaluated five dampness/mold-related factors (water stains, current moisture, rust, visible mold, and mold odor) in 50 department areas of one water-damaged hospital building and one comparison (not known to have water incursion problems) hospital building. In the study, we scored each area from 0 to 3 (none to profuse) for moisture, water stains, and rust; from 0 to 2 (none to profuse) for visible mold; and 0 to 2 (none to strong) for mold odor [31]. The study also collected health information from 834 participants in the two buildings. Total dampness/mold scores summed over the five environmental factors for each area were assigned to the participants based on the proximity of their work area to the evaluated area. This study demonstrated again that the dampness/mold score in the damaged hospital building was higher (5.5) than the comparison building (3.4) [31]. Similar to the community college study, the total dampness/mold score was significantly associated with post-hire onset asthma, building-related chest symptoms (wheezing or whistling in the chest, shortness of breath, or chest tightness), and building-related asthma symptoms in an exposure–response relationship (range of adjusted odds ratios = 2.1–2.5 for the third tertile) [30].

These two studies indicated that the observational method for dampness/mold may be used not only for environmental assessment but may also serve as a useful measure for the overall exposure assessment of microbial agents. Particularly, we demonstrated that the approach using the semi-quantitative scores reflecting the intensity of indoor dampness/mold rather than using the binary approach (presence or absence) was the most effective method to differentiate the level of water damage and the potential risk of respiratory illnesses. However, in those two studies, we did not demonstrate whether the semi-quantitative scores of dampness/mold were associated with actual (objective) measurements of microbial agents in environmental samples.

To examine the associations between observational scores of dampness/mold and quantitative analytical results in environmental samples, we conducted a study of three public schools (elementary, middle, and high) within the same school district in Maine [32]. We inspected 219 rooms in those schools using an observational assessment sheet similar to that shown in Figure 1. We also measured the relative moisture content in the walls, floors, and furniture [32]. The dampness/mold scores of each room were calculated by first averaging the scores for each of the five factors (water stain, water damage, mold odor, visible mold, and wetness) over the room components and then summing the factor-specific average scores over the five factors. In addition to observational assessment, floor dust samples were collected from a subset (*n* = 125) of the rooms assessed with the observational method. The samples were analyzed for total culturable fungi and bacteria, Gram-positive and negative bacteria, endotoxin, ergosterol, (1→3)-β-D-glucan, and muramic acid (a cell wall component of bacteria). Using these objective measurements, we created a mixed microbial exposure index (MMEI) by summing the agent-specific decile ranks of 125 samples for all eight microbial agents to have a representative index for the overall microbial contamination of the rooms. The prevalence of water stains in the schools was the highest (52%), followed by water damage (31%), mold odor (17%), visible mold (2%), and wetness (2%). The average score and prevalence of dampness/mold were the highest in the middle school (0.78 and 71%, respectively), followed by the primary (0.50, 63%) and high (0.36, 53%) schools, and were the highest in the basement rooms (1.48, 88%), followed by the rooms on the second (0.64, 71%) and first (0.45, 59%) floors. The study demonstrated that the dampness/mold scores were positively associated with continuous measurements of total, Gram-positive (GPB) and Gram-negative culturable bacteria (GNB), and MMEI. Rooms with dampness/mold scores higher than the median also had significantly higher levels of total culturable fungi and bacteria, GPB, GNB, endotoxin, muramic acid, MMEI, and maximum moisture content than those with scores lower than the median [32]. Furthermore, the observational dampness/mold scores were significantly higher in the rooms with the most recent water leakage compared to those with historical or no leakage. This study confirmed that the semi-quantitative observational scores of dampness/mold were associated with the objective measurements of microbial agents in environmental samples, and thus the observational method could be a useful tool for environmental assessment.

Based on the findings from the three studies discussed above, we created a standardized general assessment tool for dampness/mold that can be easily applied by facility managers, IH practitioners, and even teachers or students. The published studies provided a solid scientific foundation for the development of the DMAT and demonstrated a high applicability in assessing damp indoor environments. The DMAT, which consolidated multiple versions of the prototypes that were used in multiple projects over more than a decade and was beta-tested in school environments, was published on the NIOSH website in December 2018 (see the Section 3 below for the details) [28].

## 3. Materials

**Figure 2.**
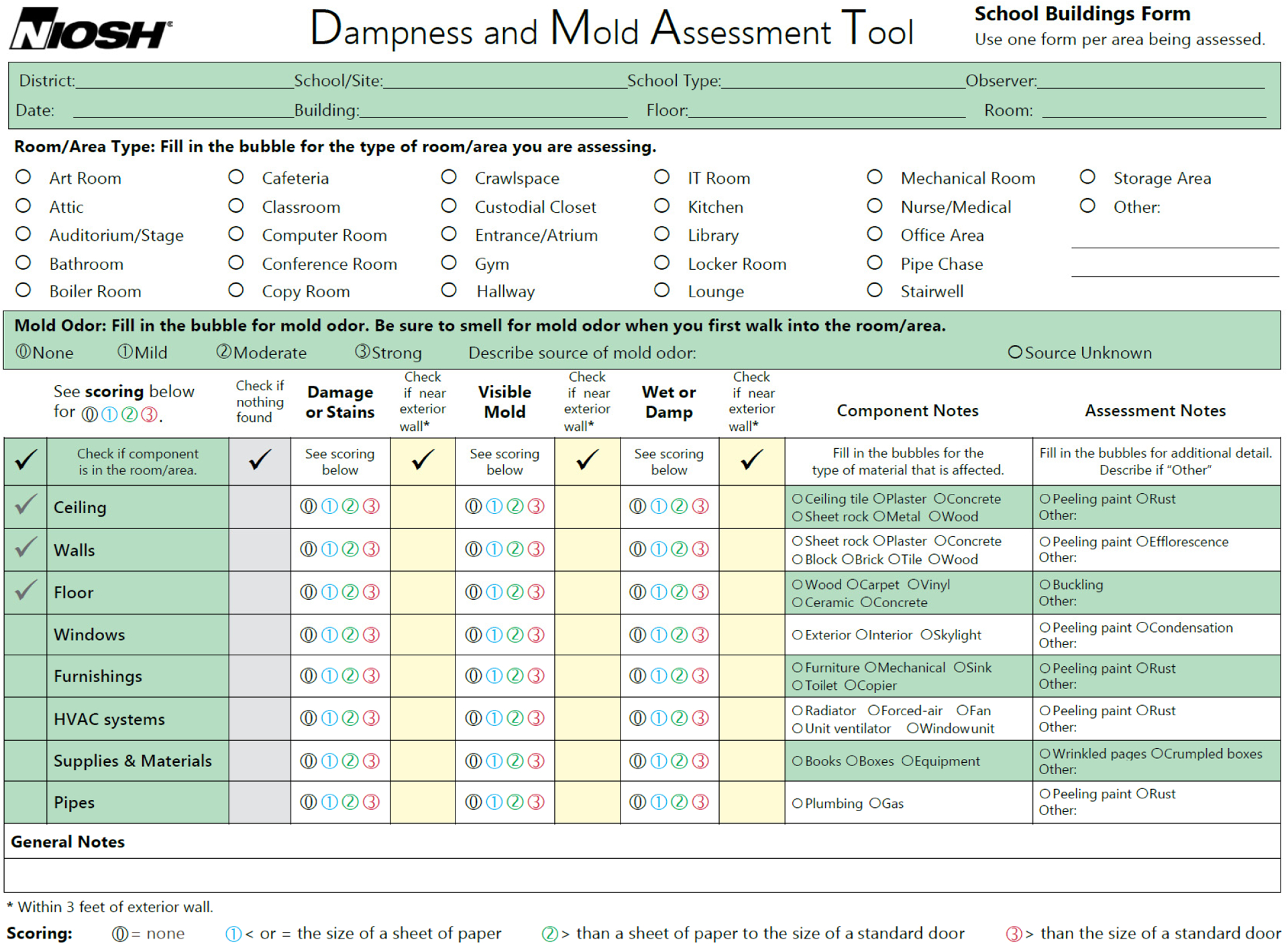
The NIOSH Dampness and Mold Assessment Tool (DMAT) for school buildings. This form can be downloaded for free from the NIOSH website: https://www.cdc.gov/niosh/docs/2019-114/pdfs/2019-114-Form-508.pdf (accessed on 10 May 2022).

**Figure 3.**
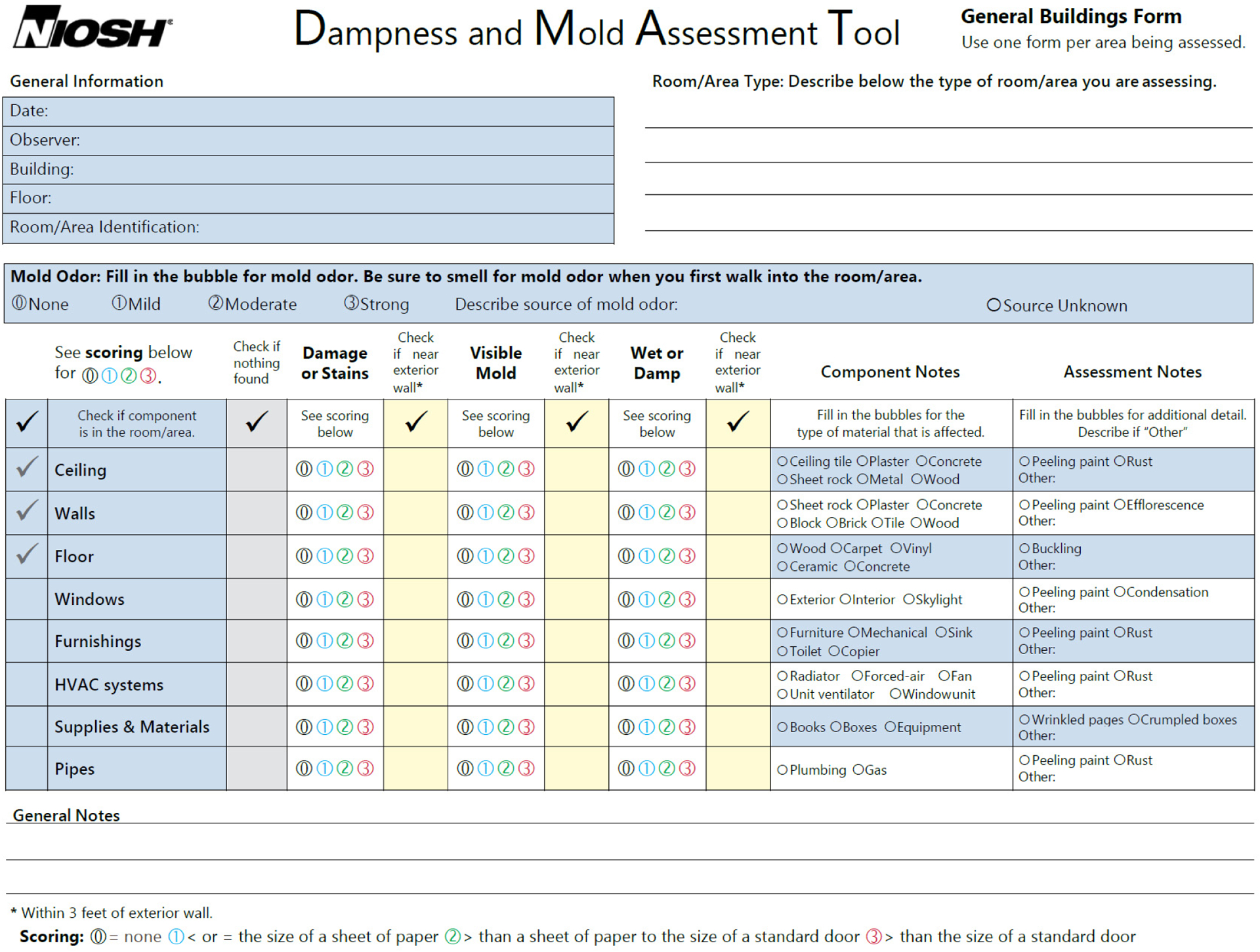
The NIOSH Dampness and Mold Assessment Tool (DMAT) for general buildings. This form can be downloaded for free from the NIOSH website: https://www.cdc.gov/niosh/docs/2019-115/pdfs/2019-115-Form-508.pdf (accessed on 10 May 2022).

There are two DMAT forms: a form for school buildings and a generalized form for all other types of buildings. These forms are presented in Figures 2 and 3. The form for school buildings was customized for collecting information about buildings, rooms, and room types in schools. Various room types that could exist in school buildings are already populated in the form for a simplified checking-off process. Information on dampness/mold-associated damage (dampness/mold-related factors) to be collected in the evaluated room/area includes mold odor, water damage/stains, visible mold, and the presence of wet or damp materials. This information should be evaluated on each of the eight room components (ceiling, walls, floor, windows, furnishings, HVAC systems, supplies/materials, and pipes). The types of material of each room component can be collected in the column “Component Notes”, and additional assessment information can be written in the column “Assessment Notes.” The next section describes in detail how the form should be filled out during an observational evaluation. The form for general buildings (other than school buildings) collects descriptive information about the evaluated building, which makes the form more generally applicable to different types of buildings, such as homes, hospitals, commercial buildings, or office buildings. The main section for evaluating dampness/mold-related factors is the same as the form for school buildings.

We also created an instructional guide for users, which is available on the NIOSH website as a free download for school buildings [33] and general buildings [34]. The instructional materials include the DMAT form and detailed information on how to determine the type of the damage and its size, which is further explained with example photos taken from water-damaged rooms. The procedure in the instructional guide is also described in the next section.

## 4. Detailed Procedures

An observational assessment of dampness/mold using the DMAT does not require the collection of any environmental samples. It solely uses a visual inspection of water damage/stains, visible mold, and wetness/dampness and an olfactory assessment of mold odor. If observers have asthma, allergies, or any other respiratory health symptoms, they should be cautious in using the tool because potential exposure during the evaluation may occur and result in the health effects discussed earlier. At the site to be evaluated, general information about the buildings and location should be collected first. Then, the observer can record information about the floor level and room/area type. For school buildings, the room/area types listed in the form can simply be checked with the appropriate box.

### 4.1. Mold Odor

Once the observer enters the room/area to be evaluated, the first thing they should do is smell for mold odor and record the findings. It is important to do this first because the observer could become acclimated to the room odor and may lose sensitivity for mold odor once they have stayed in the room for a while [35]. The mold may smell musty, earthy, damp, mushroom-like, sweet, fruity, or stale and pungent like rotting wood or paper [36]. Sometimes, it may smell like dirty and wet socks. Mold odor could be one strong piece of evidence of current mold growth, even though it may not be visible (e.g., behind the walls, above the ceiling, or under the floor carpet) [36]. If there is a mold smell, the intensity of the smell should be determined as mild, moderate, or strong. Judging the intensity may be subjective; however, it would still provide useful data of the degree of mold damage. Once the intensity of mold odor is recorded, the observer should try to search for the source of the mold odor. If the source of the smell is identified, record it; if not, check the box “source unknown,” which would indicate potential hidden mold. If the source of the smell is identified with visible mold growth, the visible mold should be independently evaluated for the factor “visible mold” (see Section 4.4. Visible Mold).

### 4.2. Room Components

Once the mold odor is evaluated, the observer should identify the applicable components of the room/area because not all components listed in the form would exist in a certain room/area. The determined room/area components should be checked on the leftmost column of the main evaluation section. The ceiling, walls, and floor are already marked because they are the basic components of an enclosed room. Windows include both internal and external ones as well as skylights. Windows could consist of glass panels, frames, and sills. Furnishings could include indoor furniture, sinks with drainpipes, printers, and copiers. An HVAC system includes all systems used for ventilation, heating, or cooling the room/area, such as unit ventilators, radiators, forced air systems, window AC units, fans, and air return and supply grilles in the room/area. Supplies and materials could include books, papers, boxes, gym equipment, kitchen supplies, and other items such as toys. Pipes include any exposed pipes in the room/area such as visible water pipes. Dampness/mold-related factors should be evaluated only for the components checked in the room/area. The identification of room components is important because the number of identified and evaluated room components could be used to calculate the factor-specific average dampness/mold score or average room/area score.

Once the existing room/area components are identified, the observer is ready to evaluate water damage/stains, visible mold, and the presence of damp/wet materials. It is important to note that the assessment requires the systematic observation of each dampness/mold-related factor on every one of the identified room components. This is best achieved by assessing all the factors for one component at a time, that is, by finishing the evaluation of the four factors for one component (e.g., the ceiling) and then moving to the next component (e.g., the walls), continuing until all the existing room components are evaluated.

### 4.3. Water Damage or Stains

Water damage could include peeling paints, efflorescence, rusty metals, and warped, deteriorated, or crumbled building materials. Stains could include discoloration caused by possible water leaks, flooding, or condensation. Next, the observer should determine the size of the damage/stains and check the score as 1 for a damaged area less than the size of a sheet of standard paper (8.5 × 11 inches), 2 for a damaged area greater than the size of a sheet of standard paper but smaller than the size of a standard interior door (32 × 80 inches), or 3 for a damaged area greater than the size of a standard interior door. The determination of the size of the damage/stains should be based on the combined size of all damages within the same component. As discussed earlier, water damage/stains are the most prevalent sign of damage related to water incursion. This information could indicate historical and current water damage. Some stains may be wet from recent or ongoing water incursion, which must be independently checked for presence of wet or damp materials as below (for the factor “wet or damp” in the form).

### 4.4. Visible Mold

Mold can include patches or spots that are colored differently than the underlying material (typically, gray, brown, or black) and appear fuzzy. If you see such mold growth, this factor should be checked. For the inexperienced, it may be challenging to determine mold growth. However, if it is suspected as mold or mold growth without a musty or earthy odor, the factor should be checked, and other experienced staff or professionals may confirm the suspected growth later. The scoring criteria for the level of damage based on the determined size of visible mold are the same as for the water damage/stains, as described above.

### 4.5. Wet or Damp (Presence of Wet or Damp Materials)

Wet or damp materials can include visible signs of unnecessary moisture, such as wetness on materials, water beads or condensation, water leaks, or flooding inside the room/area. The scoring criteria to determine the level of damage are the same as for the water damage or stains and visible mold, as described above.

### 4.6. Damage near Exterior Wall

If any of the dampness/mold-related factors on each component are within three feet of exterior walls, check the box under “check if near exterior wall.” These data could provide information about whether the water incursion is external or internal leakage.

### 4.7. Nothing Found

If any of the dampness/mold-related factors are not identified for a component, check the “nothing found” column so that “zeros” for all three factors do not need to be recorded repeatedly, which saves the observer’s time.

### 4.8. Component or Assessment Notes

Additional information, such as materials of room/area components or characteristics of water damage can also be collected. The additional information may provide useful data for monitoring and repairing the damaged areas in the room/areas.

### 4.9. When and How Frequently Do We Use the DMAT?

**Figure 4.**
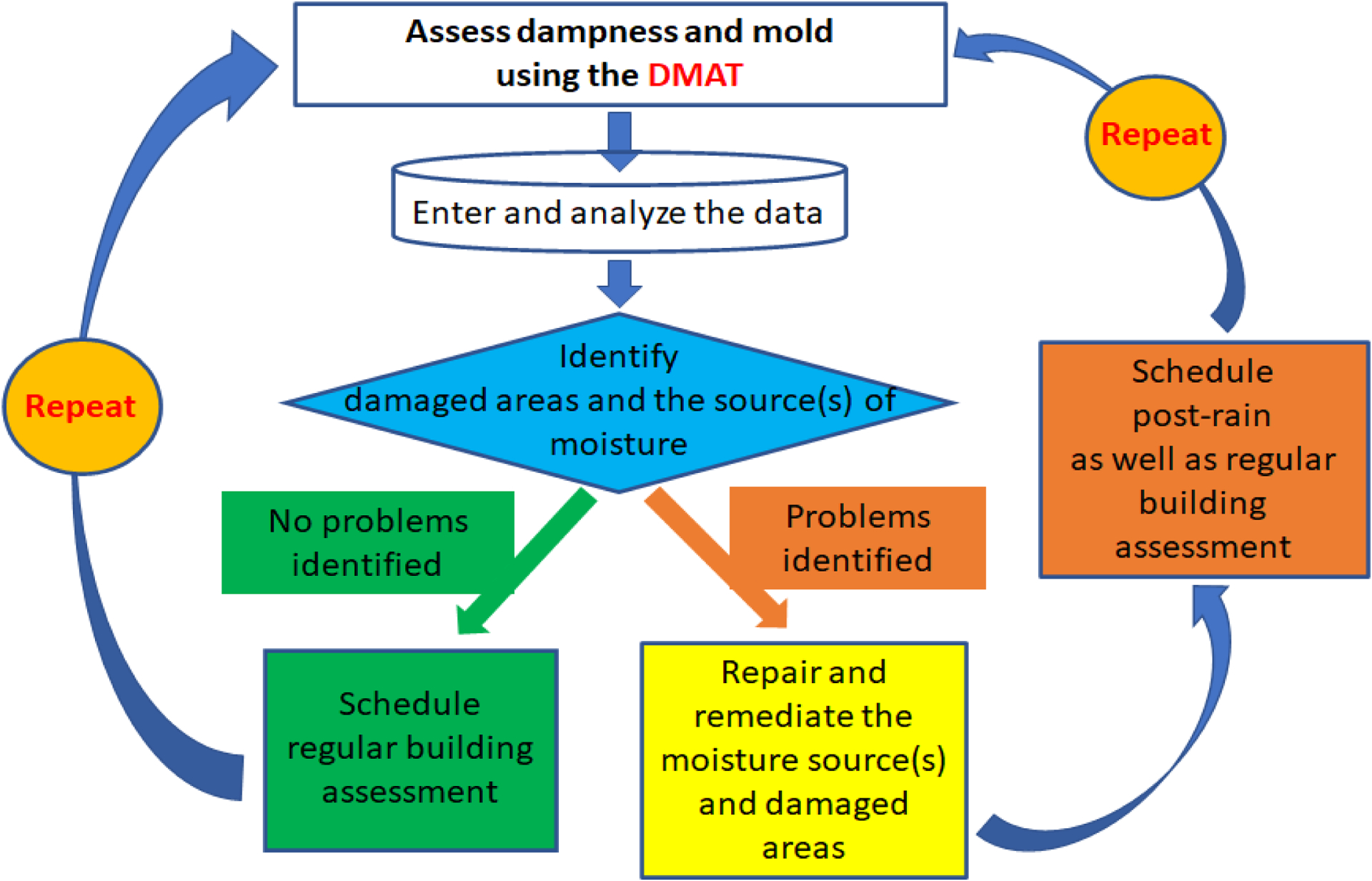
Assessment cycle of dampness and mold using the DMAT.

The NIOSH DMAT can be used to initially evaluate dampness/mold-related factors in all rooms/areas or in damage-reported rooms/areas of buildings as well as to follow up on damage in previously evaluated rooms/areas to see whether the damage is the same or getting worse. If it is getting worse (i.e., the determined or calculated damage score increased), the source of leakage or water intrusion needs to be identified and repaired. The follow-up assessments may also reveal new damage that did not exist in the previously evaluated rooms/areas. After every extreme weather event such as heavy rain, thunderstorms, or hurricanes, the DMAT can also be used to check the rooms/areas that had a history of water incursion. Thus, the evaluation cycle could include an initial assessment of dampness/mold using the DMAT, data entry and analysis, the identification of damaged areas and the sources of moisture, repair and remediation of the moisture sources and damaged areas if found, and a repeat of the regular or post-rain assessment using the DMAT (Figure 4). How often to use the DMAT may depend on regional climatic characteristics (i.e., the frequency of rain or storms, temperature, or relative humidity), but it is recommended that buildings be assessed using the tool twice a year, ideally in two different seasons, such as summer and winter.

### 4.10. Storage and Analysis of Collected Data and Interpretation of Results

**Figure 5.**
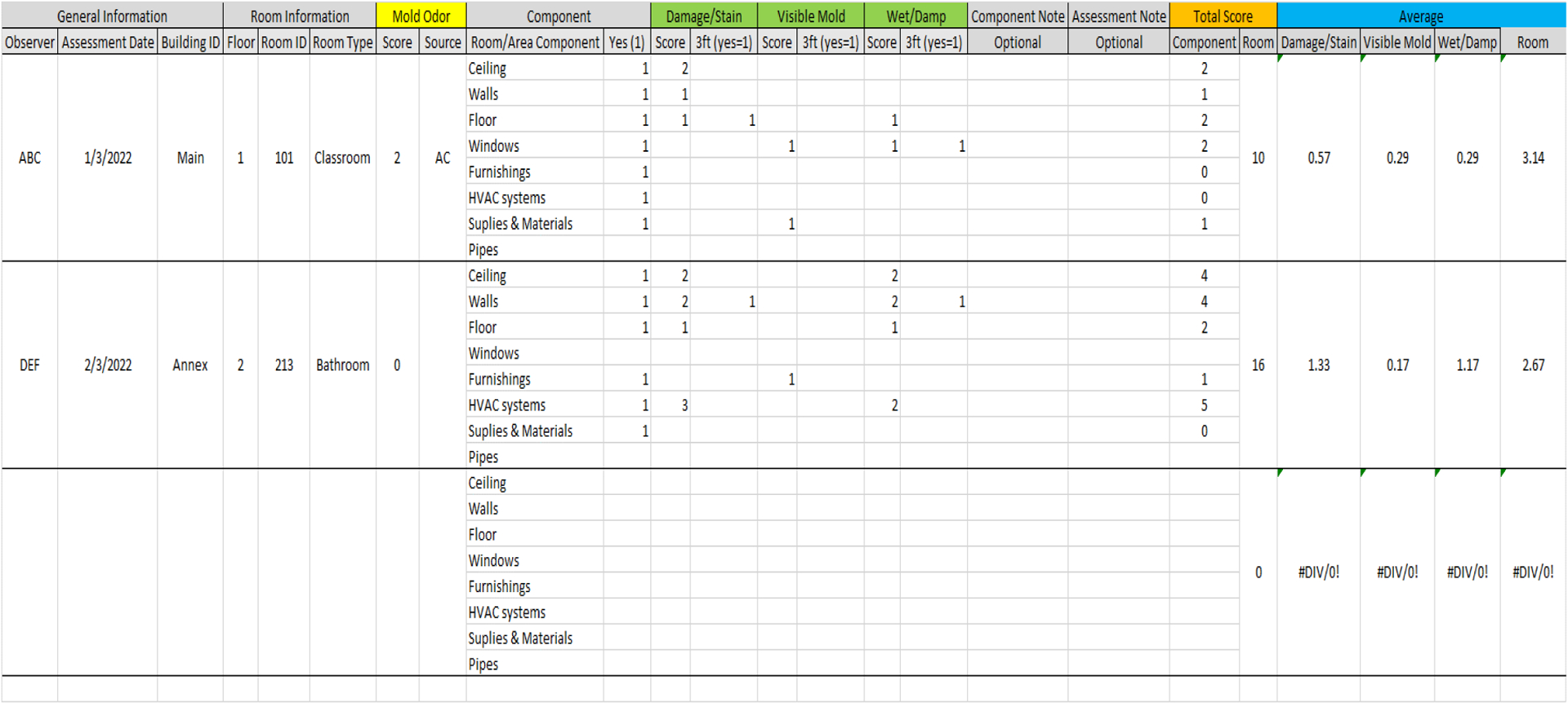
An example of a data entry form using a Microsoft Excel Sheet. The actual Excel file can be downloaded from the Supplementary Materials.

The data collected using the DMAT paper form may be keyed into a spreadsheet, such as the example shown in the Figure 5 (an actual file made using Microsoft Excel with an example data entry for two hypothetical rooms is available in the Supplementary Materials and can be downloaded for free). The spreadsheet contains the data-entry columns for all items in the DMAT form. Keyed data can be stored as an electronic data file in a computer or cloud. The score for no mold odor should be entered as “0” in the sheet. The existing room/area component should be either “1” or missing (leave as an empty cell). The determined non-zero scores for the three dampness/mold-related factors (damage/stain, visible mold, and wet/damp) should be entered in the score columns under the factor name, but “0” scores could be left missing as an empty cell. Some summary scores from the raw entered data will be automatically calculated based on a formula embedded in the sheet. In the example spreadsheet, the component-specific total score (in the sub-column heading “Component” under the column heading “Total Score” highlighted with orange in Figure 5) was calculated by adding the three dampness/mold-related factor scores (in the sub-column headings “Score” under the light-green-highlighted column headings). The total room score (in the sub-column heading “Room” under the column heading “Total Score” highlighted with orange in Figure 5) was calculated by adding all the component-specific total scores (under the column heading “Total Score” highlighted with orange in Figure 5, as described above) and the score for mold odor (in the sub-column heading “Score” in the yellow-highlighted column heading “Mold Odor” in Figure 5). We can also calculate the average score if the total score needs to be adjusted by the number of existing room components. The factor-specific average score (first three sub-columns under the blue-highlighted column heading “Average” in Figure 5) is calculated by adding the factor-specific scores (light-green highlight in Figure 5, as described above) over the existing components and then dividing it by the number of existing room components. The total average room score (the fourth sub-column heading “Room” under the blue-highlighted column heading “Average” in Figure 5) can be calculated by adding the three factor-specific average scores and the score for mold odor. To enter data from multiple rooms, the whole block for a third unlabeled room can be selected and then copied and pasted below the last recorded room. This copies and pastes all formulas written in the cells to calculate total and average scores.

The individual damage scores or the summary scores calculated within the spreadsheet can be analyzed, and the results of the analyses can be used for overseeing and maintaining damaged rooms/areas in buildings. These individual or summary scores can be compared among components or factors, or to the previously assessed individual or summary scores. These comparisons can provide essential information on what rooms/areas and components need the most attention, which would help management to more effectively allocate limited resources. In addition, the comparison of the current scores to the previous ones would help inform whether previous damage is getting worse or if there is new damage. If new damage is found, further investigation may yield additional information on the source of leakage. Any worsening damage should also be further investigated to identify potential sources of water incursion that should be remediated once the source is found. Delaying remediation could lead to worsening damage and the development of respiratory symptoms in occupants. Our previous study demonstrated that once respiratory symptoms develop they may not be easily resolved, even with remediation [37]. It is important to note that the superficial maintenance of damage (e.g., replacing ceiling or floor tiles, or painting over water stains) without repairing the sources of water incursions can result in recurring and worsening damage [32].

Dried water stains may represent historical water incursion, while wet water stains and the presence of wetness/dampness, visible mold, and mold odor could represent ongoing or recent damage. Therefore, if the presence of wet/damp building materials, visible mold, or mold odor is found, immediate actions, including investigating the potential sources of water incursion and prompt remediation if sources are identified, are required. Although the DMAT assessment does not identify all hidden mold with visual observation, mold odor without identified visible mold growth may indicate the presence of hidden mold, such as behind the walls, underneath the carpet, or above the ceiling.

Observers who complete the DMAT form do not need to have special knowledge on building engineering, environmental health science, or industrial hygiene. However, one limitation of the DMAT is that the assessment is somewhat subjective and can vary from person to person; however, if consistency in observational assessment is maintained among multiple observers, subjectivity minimally affects the validity of the observational data. For example, if multiple observers assess rooms/areas within the same buildings or schools, they can independently evaluate the same rooms/areas [29]. If there are any disagreements in observations of the same items among the observers, inter-observer agreement can be improved via iterative quality assurance processes (e.g., through iterative discussions of the observations among observers or teams to develop an agreed and common method). In addition, the developed method should be consistently employed to collect comparable observational data among the observers.

## 5. Expected Results: Examples of Application of Collected Data

### 5.1. Example 1: Analysis of Room Mold Odor and Dampness/Mold Scores by Component

**Figure 6.**
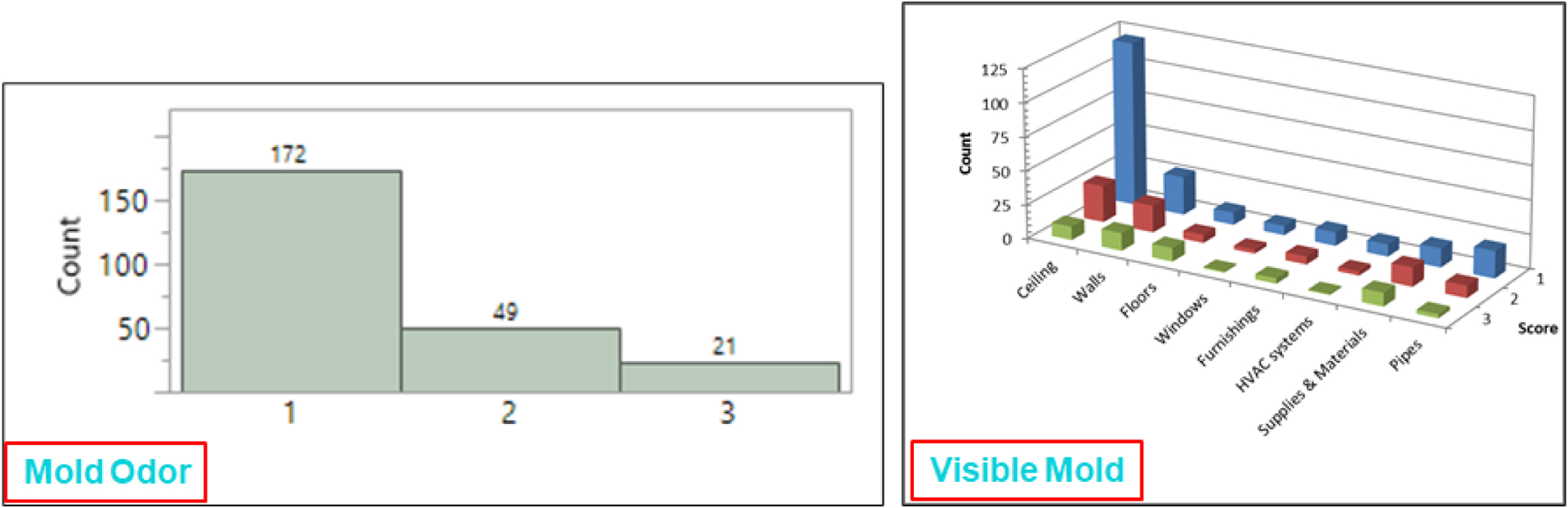

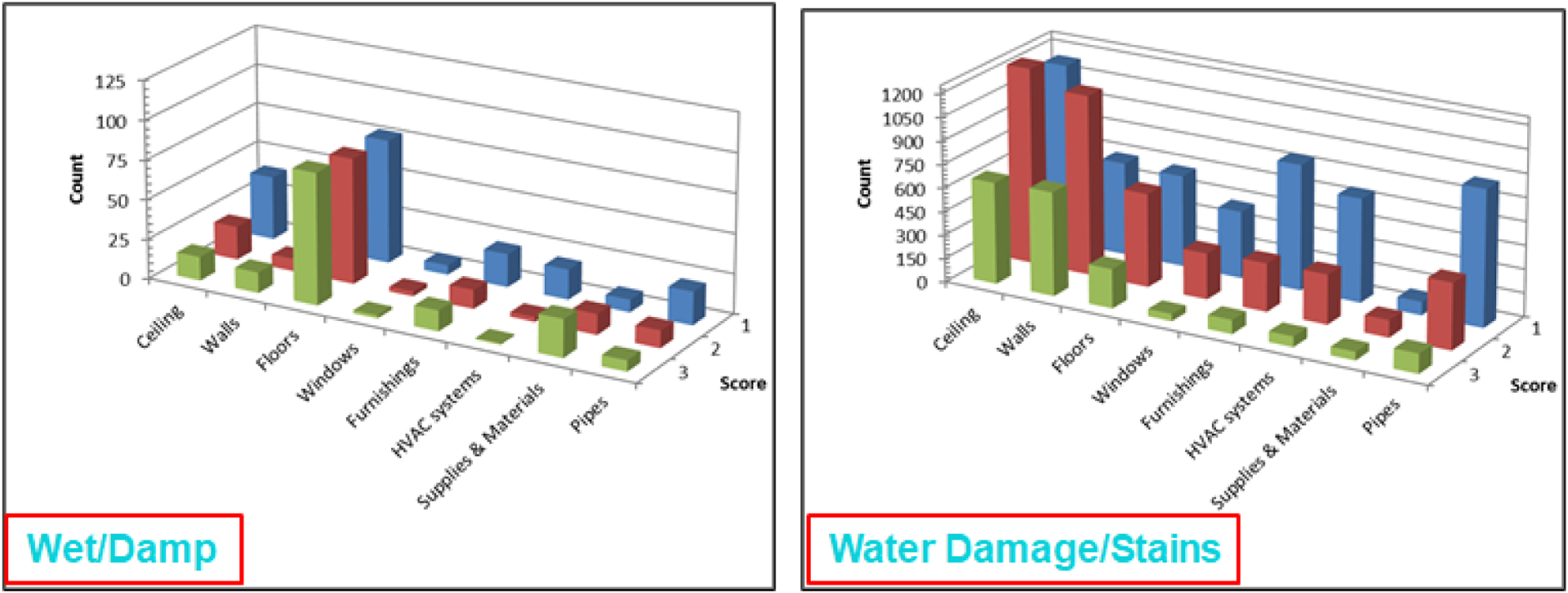
Count (frequency) of each non-zero score for mold odor, visible mold, wet/damp, and water damage/stains by room/area component.

We partnered with a school district and a teachers’ union to conduct an epidemiologic study to examine the association between exposure to dampness/mold and health among the staff of 50 elementary schools in a large city in the northeastern area of the United States [38,39]. For the study, we collected health data from school staff through a questionnaire survey and dampness/mold data using the DMAT. Five teams of two IHs evaluated dampness/mold in all accessible rooms/areas (*n* = 6492). Before the evaluation, they cross-checked their observations through the same room assessment with the DMAT and discussed the results to prepare consistent methods of observations among the teams. The collected data were analyzed, and the numbers of rooms for each non-zero score for mold odor were plotted (Figure 6). The frequencies of non-zero scores for water damage/stains, visible mold, and wet/damp materials were also plotted by room component for each score. These analyses showed which room/area components were the most damaged in the 50 schools. The analysis indicated that the ceiling and walls were the major components with visible mold or water damage/stains, implying potential external leakage from the walls or roofs. The rooms/areas with visible mold were reported to the school district for their immediate attention.

### 5.2. Example 2: Dampness/Mold Score Used for Exposure Assessment in an Epidemiologic Analysis

In the large school study described in the previous section, we summed the dampness/mold scores across the rooms/areas within a school and then averaged by the number of assessed rooms/areas to obtain an average dampness/mold score for each school. Then, each school was characterized as being above or below the median score for the 50 schools. Using this binary exposure to dampness/mold for each school, we performed logistic regressions of various respiratory symptoms adjusted for gender, race, ethnicity, age, smoking, hay fever, and mold in the home. From these analyses, we found that higher school dampness/mold scores were associated with more wheezing, chest tightness, and attacks of shortness of breath in the past 12 months (range of adjusted odds ratios = 1.32–1.61) [40]. As demonstrated, the dampness/mold score can be used to categorize health survey participants into different exposure groups for epidemiologic analyses examining the associations between exposure and health outcomes of interest. In addition, each room score can be used as an individual exposure index of dampness/mold for the occupants who spend most of their time in the room in epidemiologic analyses.

### 5.3. Example 3: Dampness/Mold Scores Used to Identify Problem Areas within the Building

**Figure 7.**
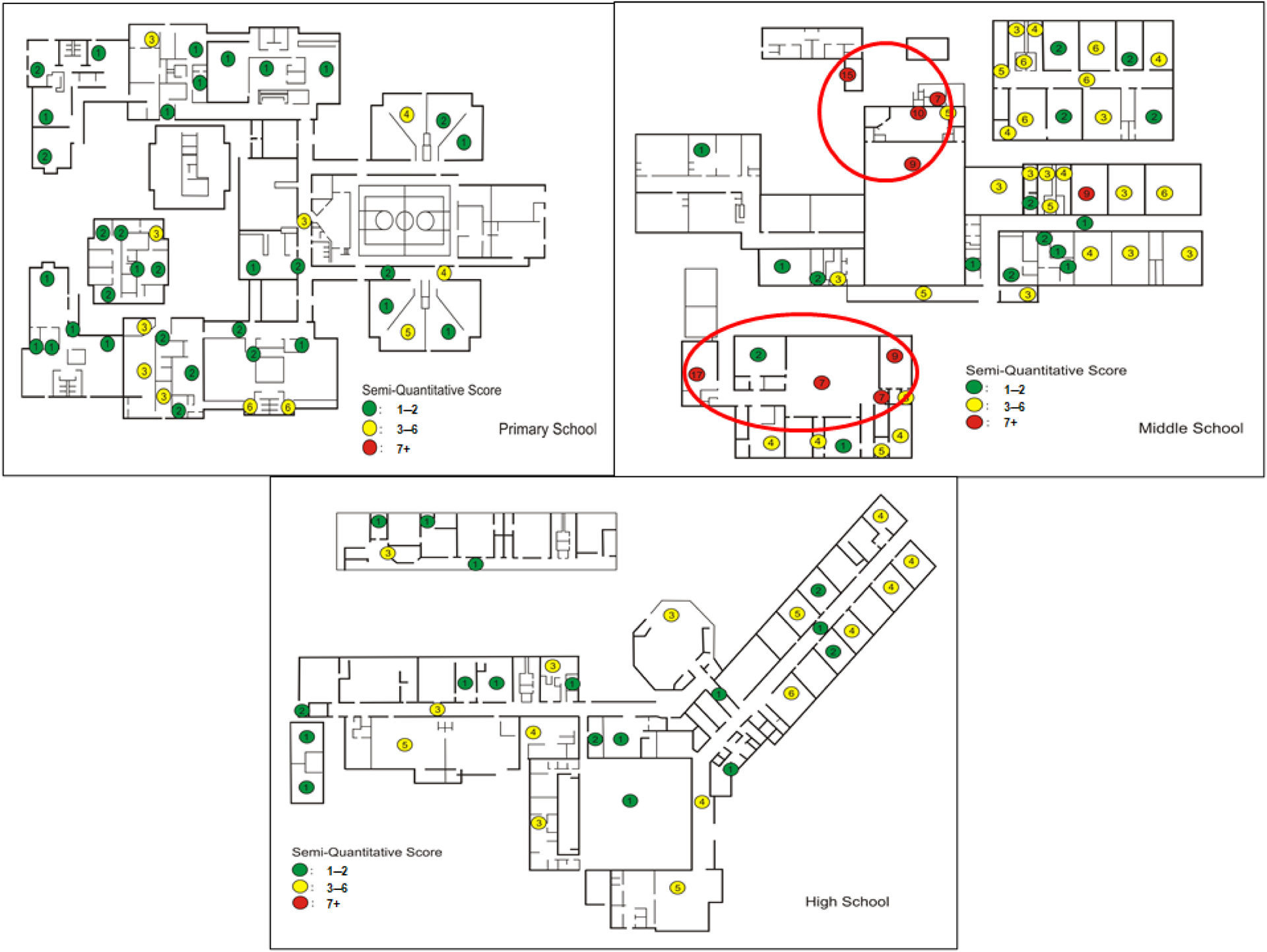
School floor maps showing the dampness and mold scores, color coded by the level of damage, with green = low, yellow = medium, and red = high. This identifies the most water-damaged areas within the school buildings.

We analyzed dampness/mold scores from the three-school study described in the experimental design section [32]. To identify the most damaged areas within the schools, we calculated room total scores, as described in the previous subsection (“storage and analysis of collected data, and interpretation of results”), and plotted the scores on school floor maps with different colors by the group of scores (green = low scores, 1–2; yellow = medium scores, 3–6; and red = high scores, 7 or higher) (Figure 7). This analysis helped us identify certain areas in the middle-school building that had the most water damage and needed further investigation. In addition, the analysis identified the problem areas in the three schools that needed to be monitored by repeating the use of the DMAT.

### 5.4. Example 4: Identifying Problem Schools and Room/Area Components in a School District

**Figure 8.**
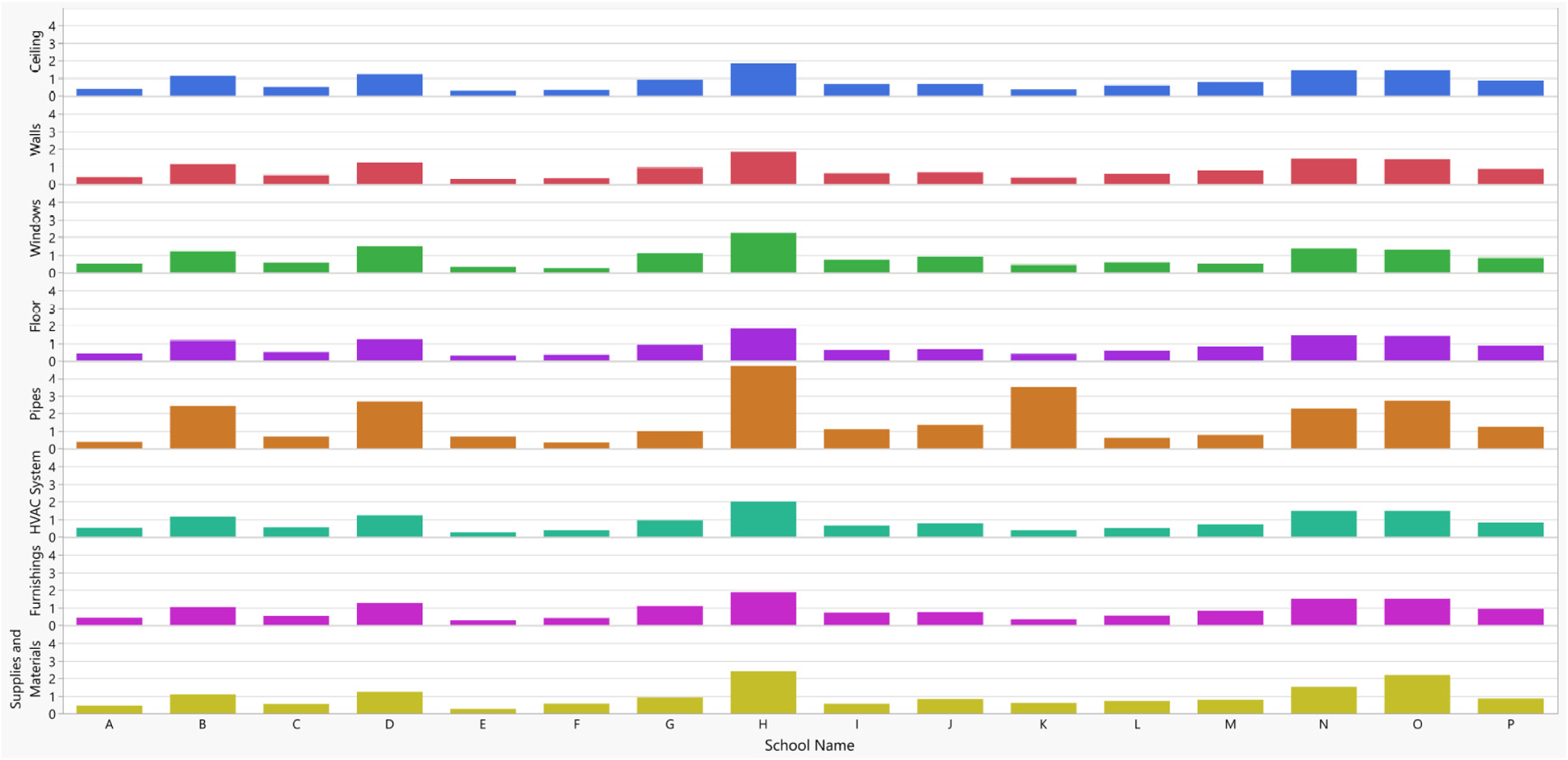
Component-specific average score for each school. The school average scores were calculated by summing all component-specific scores over all rooms/areas within a school (component-specific school total score) and then dividing the component-specific school total score by the number of rooms/areas assessed for each school.

Using the DMAT, we investigated all 2274 accessible rooms/areas in 16 schools for dampness/mold in a school district in the western United States. We analyzed the water damage/stains score because it was the most prevalent problem. The average component-specific score for each school was calculated by summing all component-specific school total scores over all rooms/areas assessed within a school (component-specific school total score) and then dividing it by the number of rooms/areas assessed. These component-specific school average scores were plotted on a bar graph by component and school (Figure 8). This analysis indicated that school H had the greatest water damage/stains among all investigated schools, followed by schools N, O, and D. Among the schools, the most water damage/stains were found in pipes followed by windows, which indicated potential water leakage from pipes or exterior walls through windows. This information helped the school district management prioritize the schools and room components that needed further investigation or repair.

## 6. Conclusions

As demonstrated, the DMAT can be a useful tool to: (1) examine associations between dampness/mold scores and health symptoms; (2) identify and record areas of dampness and mold throughout a building; (3) trigger early repair and remediation to avoid potential adverse health effects and more costly repair and remediation; (4) create awareness of potential problem areas; and (5) track past and present problem areas by repeating the use of the DMAT. Although the current article provided an example of a data entry form, users can also develop their own form for data entry and analyze the collected data in their own creative way. In the future, a mobile application may be developed. As users evaluate the rooms/areas, observations can be documented directly in the application and then transferred to a cloud-based database, which would save time and minimize data entry errors.

## Supplementary Material

DMAT Data Entry Excel Sheet
